# Repeat measures of hearing ability and ear health in the Avon Longitudinal Study of Parents and Children (ALSPAC): I. Data collected through hands-on assessment of the study offspring (G1); A resource for researchers.

**DOI:** 10.12688/wellcomeopenres.26995.1

**Published:** 2026-07-11

**Authors:** Amanda Hall, Steven Gregory, Yasmin Iles-Caven, Kate Northstone, Jean Golding

**Affiliations:** 1 Aston University Department of Audiology and Healthcare Science, Birmingham, England, B4 7ET, UK; 2 Centre for Academic Child Health, Population Health Sciences, University of Bristol Medical School, Bristol, England, BS8 2PS, UK; 3 ALSPAC, Population Health Sciences, University of Bristol Medical School, Bristol, BS1 5DS, UK

**Keywords:** ALSPAC, hearing, hearing loss, otitis media with effusion, video-otoscopy, otoacoustic emissions, audiometry, tympanometry, children

## Abstract

Acute and chronic ear problems can lead to hearing loss. Temporary and persistent hearing loss during early and mid-childhood can impact speech and language development, behaviour and other developmental milestones. There is some evidence that the consequences may be long-term in influencing outcomes related to behaviour, social interaction and mental health into adulthood. Unlike most birth cohort studies, the Avon Longitudinal Study of Parents and Children (ALSPAC) has repeated measured aspects of hearing and ear related signs and symptoms in considerable detail during childhood. This enables the identification of consequences and preventive features that can be considered. In this, the first of two Data Notes, we outline the information collected from direct assessment of the study cohort, between the ages of 8 months and 15 years. This includes features related to ear conditions and hearing ability, and indicates the variables derived and the frequencies of the results obtained during childhood and adolescence.

## Introduction

Although it is well recognised that hearing loss in young children may have long-term effects on the child ( Lieu et al., 2020), it is surprising that few of the major birth cohorts have considered hearing or otitis media with effusion in early childhood. The Avon Longitudinal Study of Parents and Children (ALSPAC) is an exception. A 10% subset of the cohort were selected for detailed examination in the first five years of life. In subsequent years, the whole cohort were invited for similar examinations of hearing. This Data Note describes the various ear and hearing measurements obtained from face-to-face assessment in the index children (G1 cohort) so that future researchers may consider the short- and long-term effects of different hearing phenotypes. We also indicate papers that have been published using this data. In a further Data Note we will describe the relevant details collected about ears and hearing by questionnaires, administered to parents, teachers and the children themselves when they were older.

## Materials and methods

### The ALSPAC cohort

With the aim of identifying features of the environment that influence the long-term physical, cognitive and psychological development of the child through adolescence and into adulthood, ALSPAC was planned to start during pregnancy and continue following the children and their parents from the time of birth onwards ( Golding et al., 2001; 2004). Pregnant women resident in Avon, UK with expected dates of delivery between 1st April 1991 and 31st December 1992 were invited to take part in the study. 20,248 pregnancies were identified as being eligible and the initial number of pregnancies enrolled was 14,541. Of the initial pregnancies, there was a total of 14,676 foetuses, resulting in 14,062 live births and 13,988 children who were alive at 1 year of age. From age 7 years, attempts were made to bolster the initial sample with eligible cases who had failed to join the study originally (n = 913). The phases of enrolment are described in more detail in the cohort profile paper and its update ( Boyd et al., 2013; Fraser et al., 2013; Northstone et al., 2025). The total sample size for analyses using any data collected after the age of seven is therefore 15,447 pregnancies, resulting in 15,658 foetuses, of which 14,901 children were alive at 1 year of age. Please note that the study website contains details of all the data that is available through a fully searchable data dictionary and variable search tool: http://www.bristol.ac.uk/alspac/researchers/our-data

**Ethical approval and informed consent**

Ethical approval for the study was obtained from the ALSPAC Ethics and Law Committee and the Local Research Ethics Committees. Informed consent for the use of all data collected was obtained from participants following the recommendations of the ALSPAC Ethics and Law Committee at the time. Participants can contact the study team at any time to retrospectively withdraw consent for their data to be used. Study participation is voluntary and during all data collection sweeps, information was provided on the intended use of data. For the majority of tests undertaken during face-to-face visits, verbal consent was obtained from participants (both parents and children as appropriate) prior to the start of any data collection. However, some tests required the completion of a written consent form. All ethical approvals can be found here: http://www.bristol.ac.uk/alspac/researchers/research-ethics/

There are two samples considered in this data note:

(a) The 10% random sample of ALSPAC selected for detailed examination from 8 months to 61 months, known as the Children in Focus.
(b) The whole cohort of ALSPAC children who were invited to face-to-face examinations of various aspects of development from 7 years onwards.

### Inclusions and exclusions

The Children in Focus cohort was chosen at random from the last 6 months of ALSPAC births, occurring between June 6th and December 11th, 1992. Excluded were those mothers who had moved away from Avon or were ‘lost to follow up’ when they moved without forwarding addresses; those who had refused to participate or fill in questionnaires; and those whose baby had died or who had two or more pregnancies in the study. Also excluded were those babies in the Avon Premature Infant Project (APIP) for very premature babies (i.e. <33 weeks gestation) and their full-term controls (a total of 52 babies out of 4257 eligible cases; 35 of these were preterm babies and 17 were controls) ( Johnson et al., 2005). There was no selection on place of residence as long as it was within the study area at the time of the first invitation to join Children in Focus. Children who moved away subsequently were still invited to participate in the face-to-face visits, although travel costs were unable to be met in full. All twins born in the eligible time period were invited to take part.

### Hands-on examinations

The aim of the hands-on assessments was to examine the children in a way that could not be done using questionnaires alone. The study children were invited to a face-to-face visit staffed by ALSPAC trained individuals specially chosen for their ability to interact positively and warmly with participants.

The ear and hearing measures that were taken during the Children in Focus visits and on the whole cohort are summarised in Table 1. All testing was overseen by qualified audiologists. Staff conducting the tests were either qualified audiologists or other staff who underwent a 3-month training period and received ongoing supervision from qualified audiologists. There was ongoing reliability checking of all ear and hearing measures including observation and repeatability checking of testing, and data reliability checking.

**Table 1.** Summary of ear and hearing face-to-face examinations.

Ear or hearing function assessed	Measure	Children in Focus (age in months at which measure taken)	Whole cohort (age in years at which measure taken)
Middle ear function	Tympanometry	8, 12, 18, 25, 31, 37, 43, 49, 61	7, 9, 10, 11, 12
	Screening acoustic reflex	Not measured	7, 11
	Video otoscopy	Not measured	9
Hearing threshold	Pure tone audiometry	61	7, 9, 11, 15
	Word recognition threshold test	31, 43, 61	Not measured
Cochlear function	Transient evoked otoacoustic emissions	Not measured	9, 11
Comfortable listening level	Typical listening level for music	Not measured	11

### Measures of middle ear function

*Tympanometry*

Tympanometry is a quick, non-invasive, objective and effective assessment of the function of the middle ear ( BSA, 2026). It measures the mobility of the eardrum (tympanic membrane) and middle ear pressure. It is not a test of hearing but provides an indication of the presence of middle ear fluid, or otitis media with effusion (OME), which may be associated with a mild/moderate hearing loss ( Cai and McPherson, 2017).

Tympanometry was carried out at all nine Children in Focus visits, and at five time points between the ages of 7 and 12 years on the whole cohort. Testing was performed using a calibrated Kamplex AT2 tympanometer for all Children in Focus examinations (i.e. 8–61 months) and the 7-year visit. For later visits a Kamplex KA9 tympanometer was used. The resultant graph (tympanogram) was visible to the parent, but staff made no attempt to interpret it at the time of the visit. The tympanograms were later classified by qualified audiologists according to the classification shown in Table 2, based on Fiellau-Nikolajsen’s modification of Jerger’s classification ( Fiellau-Nikolajsen, 1983). From 9 years onwards, middle-ear compliance and pressure (MEC and MEP), ear canal volume and grading were also recorded, and these data are also available.

**Table 2.** Tympanogram classification (per ear).

Type	Description	Classification criteria
Type A	Normal middle ear function	MEP of +100 to -100 mm H2O; peaked trace
Type C1	Indicates slight Eustachian tube	MEP of −100 to -200 mm H2O; peaked trace
Type C2	Indicates Eustachian tube dysfunction	MEP of −200 to -300 mm H2O; peaked trace
Type B	Indicates middle ear fluid or otitis media with effusion	MEP not measurable; flat trace with normal ECV
Type P	Indicates perforation	MEP not measurable; flat trace with large ECV
Type G	Indicates grommet in-situ	MEP not measurable; flat trace with large ECV; report of grommet insertion
Type U	Unclassified	None of above

MEP: Middle ear pressure; ECV: Ear canal volume.

Table 3 shows the names of the relevant variables available in the ALSPAC resource. Table 4 shows the proportion of those attending the visits who had a recordable tympanometry result. Good coverage was achieved at most visits, except for the 12-year visit when limited funding resulted in curtailment of tympanometry testing.

**Table 3.** Tympanometry timepoints and variable names.

Sample	Age	Variable names
Children in Focus	8 months	cf500, cf510a, cf510b, cf520, cf530
	12 months	cf501, cf511a, cf511b, cf521, cf531
	18 months	cf502, cf512a, cf512b, cf522, cf532
	25 months	cf503, cf513a, cf513b, cf523, cf533
	31 months	cf504, cf514a, cf514b, cf524, cf534
	37 months	cf505, cf515a, cf515b, cf525, cf535
	43 months	cf506, cf516a, cf516b, cf526, cf536
	49 months	cf507, cf517a, cf517b, cf527, cf537
	61 months	cf508, cf518a, cf518b, cf528, cf538
Whole cohort	7 years	f7hs051, f7hs060, f7hs061, f7hs062, f7hs063
	9 years	f9hs200, f9hs203, f9hs205, f9hs216
	10 years	fdba144, fdba145, fdba146, fdba147, fdba149, fdba150, fdba151, fdba152
	11 years	fehs600, fehs601, fehs602, fehs603, fehs604, fehs605, fehs606, fehs607, fehs608, fehs609, fehs612, fehs613, fehs614, fehs615, fehs616, fehs617
	12 years	ff2150, ff2151, ff2152, ff2160, ff2161, ff2162, ff2163, ff2164, ff2170, ff2171, ff2172, ff2173, ff2174

**Table 4.** Number of those with tympanometry results available and the proportion indicative of otitis media with effusion (bilateral or unilateral B type tympanogram).

Sample	Age	N of cases	Bilateral OME (%)	Unilateral OME (%)
Children In Focus	8 months	1183	24.9	19.5
	12 months	1065	16.0	20.4
	18 months	1052	23.6	18.2
	25 months	995	9.5	12.3
	31 months	1061	13.3	13.8
	37 months	1016	9.0	9.2
	43 months	1039	11.8	14.8
	49 months	961	5.9	10.4
	61 months	949	7.2	9.6
Whole cohort	7 years	7817	2.2	5.1
	9 years	7423	1.2	3.1
	10 years	6148	1.4	3.0
	11 years	6692	1.1	3.1
	12 years	2082	1.5	3.9

OME: otitis media with effusion.

There was a reduction in the proportion of children who had tympanograms indicating bilateral OME over time, from 24.9% at 8 months to 7.2% at 61 months (5 years). By age 12, only 1.5% of children had evidence of bilateral OME.

Publications using the OME results include: (a) A study documenting the pattern of OME using tympanometry in each child up to age 5 ( Midgley et al., 2000). This showed that there was a dramatic effect of season, with increased prevalence of OME in the winter months and gradually decreasing prevalence as the child got older; (b) A study of the number of timepoints at which the child had OME. This indicated a trend for lower IQ scores with increased OME occasions and showed a diminished deficit if the child had been cognitively stimulated by the parents ( Hall et al., 2014); (c) A study examining the educational impact of co-occurring mild visual and hearing impairment. This showed a cumulative impact of dual sensory impairment ( Hill et al., 2019); (d) A study demonstrating increased prevalence of OME if the child attended Day Care with other children ( Dewey et al., 2020).

*Screening acoustic reflex*

Acoustic reflex testing measures the response of the middle ear stapedius muscle and auditory neural system to sound ( BSA, 2026). It provides a measure of the function of the middle ear muscles and VII and VIII cranial nerve function. Presence of an acoustic reflex is consistent with normal middle ear and brainstem function. Absence of an acoustic reflex could indicate a middle ear problem, hearing loss or disruption of neural function.

Screening ipsilateral acoustic reflexes were measured at the 7 and 11-year visits after tympanometry (in those with no indication of middle ear effusion). At age 7, it was measured using a 1 kHz stimulus at stimulus levels of 80 and 100 dB. At age 11, it was measured using a 1 kHz stimulus at 85, 95 or 105 dB. Presence or absence of a response at each level was noted ( Table 5).

**Table 5.** Acoustic reflex timepoints, variable names and summary results.

Sample	Age	Variable names	N of measures, right ear	Absent reflex, right ear, maximum stimulus level (%)	N of measures, left ear	Absent reflex, left ear, maximum stimulus level (%)
Whole cohort	7 years	f7hs1000, f7hs1001, f7hs1002, f7hs1003, f7hs1004, f7hs1005	6624	18	6546	17
	11 years	fehs700, fehs701, fehs702, fehs703, fehs704	5846	13	7206	10

Results show around 10–13% of children at age 11 had absent acoustic reflexes. The significance of these findings is unclear and could indicate subtle middle ear disorders, sensory or neural hearing loss.

*Video otoscopy*

Video otoscopy involves taking a photographic image of the tympanic membrane, and allows abnormalities to be detected, such as retractions, perforations or middle ear effusion. Video otoscopy was carried out at the 9-year visit. Pictures were taken of each child’s left and right ear drum using a hand-held camera probe. Two pictures were taken of each ear drum: one of the attic region (pars flaccida), and one of the lower section (pars tensa). Each child was given a picture of their ear drum to take home. Pictures were stored electronically and were analysed after the visit by two consultant Ear, Nose and Throat Surgeons.

The pars flaccida and pars tensa were categorized as either normal or abnormal. Those that were categorized abnormal were then coded within the following major categories: 1) atelectasis or retraction of the pars tensa; 2) tympanosclerosis of the pars tensa; 3) perforation of the pars tensa; 4) retraction of the pars flaccida; 5) attic crust/cholesteatoma; 6) middle ear effusion, and 7) presence of ventilation tubes (see Maw et al., 2011 for further details). Table 6 summarises the results.

**Table 6.** Video-otoscopy time point, variable name and summary results.

Sample	Age	Variable name	N of measures	Abnormal tympanic membrane, right ear (%)	Abnormal tympanic membrane, left ear (%)
Whole cohort	9 years	pt_nml_l, pf_n_l, pt_nml_r, pf_n_r	6866 *	34.2	36.1

* excluding duplicate pictures.

Publications documenting the video otoscopy results include: (a) A study examining the frequency of middle ear disease ( Maw et al., 2011); (b) A study to determine which factors are associated with middle ear disease ( Clamp et al., 2020).

### Measures of hearing threshold

*Pure tone audiometry*

Pure tone audiometry gives a measure of hearing threshold level and can be tested using headphones to measure air conduction thresholds and a bone vibrator to measure bone conduction thresholds. Pure tone audiometry enables classification of the degree and type of hearing loss in each ear. Audiometry was carried out at the final Children in Focus visit at age 61 months, and at the visits at age 7, 9, 11 and 15 years (variable names in Table 7).

**Table 7.** Pure tone audiometry timepoints and variable names.

Sample	Age	Type of audiometry and frequencies tested	Variable names
Children in Focus	61 months	Air conduction: 0.5, 1, 2, 4, 8 kHz Bone conduction: 0.5, 1, 2, 4 kHz	cf541, cf541A, cf541B, cf542, cf542A, cf542B, cf543, cf543A, cf543B, cf544, cf544A, cf544B, cf545A, cf545B
Whole cohort	7 years	Air conduction: 0.5, 1, 2, 4, 8, 16 kHz Bone conduction: 1, 4 kHz	f7hs009, f7hs010, f7hs011, f7hs012, f7hs013, f7hs014, f7hs015, f7hs016, f7hs017, f7hs018, f7hs020, f7hs021, f7hs022, f7hs023, f7hs024, f7hs025, f7hs027, f7hs028, f7hs030, f7hs031
	9 years	Air conduction: 0.5, 1, 2, 4, 8, 16 kHz Bone conduction: 0.5, 1, 4 kHz	f9hs009, f9hs010, f9hs011, f9hs012, f9hs013, f9hs014, f9hs015, f9hs016, f9hs017, f9hs018, f9hs020, f9hs021, f9hs022, f9hs023, f9hs024, f9hs025, f9hs027, f9hs028, f9hs030, f9hs031, f9hs032
	11 years	Air conduction: 0.5, 1, 2, 3, 4, 6 8 kHz Bone conduction: 0.5, 1, 2 kHz	fehs404, fehs405, fehs406, fehs407, fehs408, fehs409, fehs410, fehs411, fehs412, fehs413, fehs414, fehs415, fehs416, fehs417, fehs418, fehs419, fehs420, fehs421, fehs422
	15 years	Air conduction: 1, 4 kHz	fh4120, fh4121, fh4130, fh4131

At all ages, audiometry was performed as per British Society of Audiology (BSA) standards at the time with thresholds taken as 2 out of 3 presentations on the ascending scale ( BSA, 2026). Testing started with air conduction audiometry, then moved to bone conduction. From age 9, the first ear tested was randomised (according to whether the clinic ID number was odd or even) across children. Audiological technicians and staff specifically trained in audiology carried out the audiometry tests in a sound treated room or booth. The reliability of each staff member’s audiometry testing was regularly assessed.

At age 7, a Kamplex AD12 audiometer (type TDH39 headphones) or a GSI 61 audiometer (type TDH50P headphones) was used. At ages 9, 11 and 15 a GSI 61 clinical audiometer with TDH50P headphones was used. A B71 bone vibrator was used at each visit with all audiometers. HAD2000 headphones were used with the GSI61 audiometer for measuring 16 kHz thresholds. All equipment was regularly calibrated following guidance in BSA ( BSA, 2026), and the equipment number and calibration record relating to each child was noted with their results. Non-standard testing conditions were recorded.

At the start of the session, parents were informed that they would not be told any details of their child’s test results, but they would be informed if their child’s hearing was satisfactory or whether a referral was recommended. Table 8 summarises audiometry hearing threshold results.

**Table 8.** Frequency of those who had pure tone audiometry measured at each visit and whether hearing loss was present at 4 kHz.

Sample	Age	Variable names	N of measures, right ear	Hearing loss >20 dB right ear at 4 kHz (%)	N of measures, left ear	Hearing loss >20 dB left ear at 4 kHz (%)
Children in Focus	61 months	cf544a cf544b	926	9.1	940	9
Whole cohort	7 years	f7hs013 f7hs023	7763	4.7	7761	4.9
	9 years	f9hs013 f9hs023	7373	2.6	7370	3.2
	11 years	fehs408 fehs416	7072	2.1	7068	2.5
	15 years	fh4120 fh4130	4733	1.8	4734	2. 8

Publications documenting a detailed examination of the audiometry data include: (a) A study which examined the prevalence of mild and high-frequency bilateral sensorineural hearing loss at age 7–11 years, giving a combined prevalence of 0.5% (95% CI 0.4–0.8%) ( Hall et al., 2011); (b) A study where the hearing results were used as outcomes of a randomised trial of delayed surgery (watchful waiting) for glue ear ( Hall et al., 2009). In addition, a number of publications have explored genetic associations with hearing thresholds ( Bitner-Glindzicz et al., 2009; Hall et al., 2012; Harrison et al., 2015; Perrino et al., 2020; Rodriguez et al., 2009; Schmitz et al., 2021). Publications examining the relationship between hearing loss and speech and language include Bornstein et al., 2018; Carding et al., 2006; Wren et al., 2016.

*Word recognition threshold test*

The Automated McCormick Toy Test measures the word recognition threshold (a threshold measure of hearing-for-speech) ( Ousey et al., 1989; Palmer et al., 1991). Trained staff in a sound treated room used the IMATT (Institute of Hearing Research McCormick Automated Toy Test) to assess the child’s word recognition threshold using the Children in Focus sample of children at 31, 43 and 61 months of age ( Table 9). The test results were recorded as the word discrimination threshold in decibels.

**Table 9.** Word recognition threshold test timepoints, variable names and summary data.

Sample	Age	Variable names	N of cases	Mean threshold in quiet (SD)
Children in Focus	31 months	cf553	976	31.6 dB (8.3)
	43 months	cf563	1044	26.6 dB (7.3)
	61 months	cf572	975	25.5 dB (6.2)

At the 43-month examination, parents were informed if their child’s test result was greater than 35dBA.

The publication documenting the word recognition threshold and the impact of unilateral and bilateral OME on hearing ability for speech is Hall et al., (2007).

### Measures of cochlear function

Otoacoustic emissions give a measure of cochlear function ( Kemp, 2002). Transient evoked otoacoustic emissions were recorded at the cohort visits at ages 9 and 11, using the Otodynamics ILO92 system. Click stimuli were presented at a gain of −10.5 dB and − 19.5 dB (re: reference click at ~80 dB sound pressure level (SPL)) using a standard adult probe. Recordings were made in the linear mode. These settings were used as lower-level stimuli may be more sensitive to changes and differences in cochlear function.

The test was only performed with children where otoscopy showed no sign of infection or wax blockage. The child sat in a Bilsom sound-attenuating booth and was given scripted verbal instructions to remain still. Nonstandard testing conditions were recorded. The left ear was tested first, and then the right.

Analysis of the OAE waveforms was performed at the Institute of Sound & Vibration Research, University of Southampton (see Hall et al., 2012 for further information). Analysis of the OAE waveforms concentrated on the measure that is conventionally named as response, which is the SPL of the recorded components that are common to the two interleaved averages obtained during recording, conventionally denoted by A and B. The response measure was obtained from the raw (unfiltered) recordings and also after filtering into frequency bands centred on 1, 2, 3 and 4 kHz. Each filter had a bandwidth of 1 kHz. Table 10 shows the relevant variables and a sample of summary data.

**Table 10.** Otoacoustic emissions timepoints, variable names and summary response level data.

Sample	Age	Variable names	N of measures, right ear	OAE response dB 4 kHz right ear Mean (SD)	N of measures, left ear	OAE response dB 4 kHz left ear Mean (SD)
Whole cohort	9 years	f9oa303, f9oa307, f9oa311, f9oa315, f9oa319, f9oa323, f9oa327, f9oa331, f9oa335, f9oa339	5469	2.76 (7.4)	5688	2.56 (7.4)
	11 years	fehs513, fehs517, fehs521, fehs525, fehs529, fehs533, fehs537, fehs541, fehs545, fehs549	4645	2.24 (7.5)	4933	1.92 (7.4)

Publications where otoacoustic emission data were examined include Hall et al. (2012) and Harrison et al. (2015).

### Measures of comfortable listening level

An objective measure of the child’s comfortable listening level was measured when the children were age 11 ( Table 11). They were played a short piece of music through a portable CD player and supra-aural, Sennheiser PX40 headphones. They were asked to set the CD player at the volume level at which they would typically listen to music (run 1), and then this was repeated (run 2).

**Table 11.** Comfortable listening level timepoint and variable names.

Sample	Age	Variable names	N of measures (run 1)	Mean volume setting (SD, range) Run 1	N of measures (run 2)	Mean volume setting (SD, range) Run 2
Whole cohort	11 years	fehs803 fehs804	4421	9.2 (5.9) Range 1–25	4417	8.81 (5.9) Range 1–25

The music was composed specifically for the purpose and thus had not previously been heard by the children. The sound pressure level of the CD player and headphones was calibrated by ISVR, Southampton University using a KEMAR manikin (head and torso simulator) to give the equivalent sound pressure level at the eardrum for each volume setting. The minimum volume setting of the CD player was 1, and the maximum was 25. At a volume level of 25, the A-weighted sound level at the eardrum was 85–86 dB A. Each increment on the volume setting was equivalent to a change of 0.9–1.2 dB.

The listening levels of the cohort have been documented in Hall et al. (2020), in which the volume level set by the child was noted and a binary variable representing the highest 10% of scores as a measure of tolerance to loud sounds was identified, with the expectation that those who are intolerant to loud sounds would choose a lower volume level on the headphone test.

## Summary

This data note has provided an overview of the physical ear and hearing measures that have been taken from the ALSPAC G1 cohort. Key outputs to date provide prevalence data on a range of hearing measures throughout childhood and have examined the impact of otitis media in childhood on developmental outcomes. The data should be useful for researchers who investigate causes of hearing loss, the impact of sensory function on development and later outcomes in adulthood.

## Data Availability

ALSPAC data access is through a system of managed open access. The steps below highlight how to apply for access to the data included in this paper and all other ALSPAC data. Note that variable names are included in the tables within this paper. Please read the ALSPAC access policy ( http://www.bristol.ac.uk/media-library/sites/alspac/documents/researchers/data-access/ALSPAC_Access_Policy.pdf) which describes the process of accessing the data and biological samples in detail, and outlines the costs associated with doing so.
1. You may also find it useful to browse our fully searchable research proposals database ( https://proposals.epi.bristol.ac.uk/), which lists all research projects that have been approved since April 2011. 2. Please submit your research proposal ( https://proposals.epi.bristol.ac.uk/) for consideration by the ALSPAC Executive Committee using the online process. You will receive a response within 10 working days to advise you whether your proposal has been approved. If you have any questions about accessing data, please email: alspac-data@bristol.ac.uk (data) or bbl-info@bristol.ac.uk (samples). The ALSPAC data management plan ( http://www.bristol.ac.uk/media-library/sites/alspac/documents/researchers/data-access/alspac-data-management-plan.pdf) describes in detail the policy regarding data sharing, which is through a system of managed open access. Ethics – see Methods.
