# Supplementary material for: Repeat measures of hearing ability and ear health in the Avon Longitudinal Study of Parents and Children (ALSPAC): I. Data collected through hands-on assessment of the study offspring (G1); A resource for researchers
Source: Wellcome Open Res. 2026 Jul 11;11:433. [Version 1] doi: 10.12688/wellcomeopenres.26995.1 (PMC13591046; doi:10.12688/wellcomeopenres.26995.1)
Supplement: Peer review history

# Reviewer report: Reviewer response for version 1

**DOI:** 10.21956/wellcomeopenres.29716.r166494

**Published:** 2026-09-21

**Authors:** Karl R. White

**Affiliations:** 1 Utah State University, Logan, UT, USA

This is an excellent article describing the Avon Longitudinal Study of Parents and Children with a focus on the information it contains regarding repeated measured aspects of hearing and ear related signs and symptoms. The data base is described well  and the publications that have been based on this data set are interesting and relevant. How other researchers can gain access to it for additional analyses is well done. I was not aware of this resource and suspect that is true for many other people who are conducting research related to childhood hearing loss. Increased awareness of the availability of this data set would be good. The only suggestion I have is that it would have been nice if the information in Table 8 had been split into two tables with one for children with fluctuating hearing loss and the other for children with permanent hearing loss.

Are sufficient details of methods and materials provided to allow replication by others?

Yes

Is the rationale for creating the dataset(s) clearly described?

Yes

Are the datasets clearly presented in a useable and accessible format?

Yes

Are the protocols appropriate and is the work technically sound?

Yes

Reviewer Expertise:

Identification and treatment of childhood hearing loss

I confirm that I have read this submission and believe that I have an appropriate level of expertise to confirm that it is of an acceptable scientific standard.

# Reviewer report: Reviewer response for version 1

**DOI:** 10.21956/wellcomeopenres.29716.r162056

**Published:** 2026-08-07

**Authors:** Petroc Sumner

**Affiliations:** 1 Cardiff University, Cardiff, UK

This brief article explains the audiological data available in the ALSPAC dataset. it does so with a good balance of detail and conciseness. Although the ALSPAC data has its own website and listings, it can be quite hard to navigate and most researchers would not know to look there for longitudinal audiology data. Therefore this data note serves and important and useful purpose. 

 One comment. The authors say that they will write another note describing the questionnaire data relevant to hearing. There is also data in ALSPAC from questions asked in clinic (rather than measures taken) for example on hyperacusis. I am not sure where this fits, as it is not included here, but neither is it part of the remote questionnaire sets in ALSPAC. it would be useful to clarify.

Are sufficient details of methods and materials provided to allow replication by others?

Yes

Is the rationale for creating the dataset(s) clearly described?

Yes

Are the datasets clearly presented in a useable and accessible format?

Yes

Are the protocols appropriate and is the work technically sound?

Yes

Reviewer Expertise:

sensory

I confirm that I have read this submission and believe that I have an appropriate level of expertise to confirm that it is of an acceptable scientific standard.
